# Analgen

**Published:** 1893-09-09

**Authors:** 


### NEW DRUGS AND PREPARATIONS.

[We should be glad to receive, at our Office, 428, Strand, London, W.C., from the manufacturers, specimens of all new preparations and drugs which may be brought out from time to time.]

### ANALGEN (*concluded*).

In connection with a case of tabes dorsalis, with tightening pains, to whom we administered analgen, the reports of Dr. Goliner, of Erfurt, have considerable interest (Reich's "Medicinal Auzeiger," 1893, No. 4), from which we quote:—

"Dr. Vis, of Freiburg, has recently succeeded in producing a derivative of quinoline, viz., analgen, which seems destined to act as a first-rate nervine. The more or less severe pains which occur in various nerve diseases certainly justify in searching after new, reliable anti-neuralgic remedies. True, our medical treasury is not exactly poor in such remedies; we may mention antipyrine, phenacetine, exalgin, &c. However, every medical man, probably, knows by experience that many nervous troubles obstinately resist these remedies, and in those cases where real effect has once been obtained the case becomes complicated with all manner of disagreeable symptoms, often such as collapse, inordinate perspirations, cyanosis, palpitations, and the like. We therefore welcome any remedy which, without being subject to the said ill effects, acts promptly as an anti-neuralgic. The chemical factory of Dahl and Co., in Barmen, some time ago, placed a quantity of analgen at my disposal for experimental purposes. I have employed the same for typical neuralgia, tabes dorsalis, and migraine, and with what result may be seen from the following reports of cases:—

"1. A merchant, 36 years old, without syphilitic taint, suffered two years since from excruciating pains in the right thigh. Later attacks of pain also occurred about the stomach, which were combined with visceral neuralgia and with incontinentia alvi. The other symptoms were those common to well-marked tabes dorsalis. There was also failure of the plantar reflex, of the tendon reflexes and slight ataxy in the upper extremities. The eyes showed myosis and reflective rigidity of the pupils. The treatment with the faradic pencil certainly improved the visceral neuralgia after a few weeks, but the effect was only temporary. The patient took morning and evening a gramme of analgen, and in three days felt a decided improvement; the feeling of pain in the waist and the stinging pains disappeared altogether, after the patient had taken two grammes analgen daily for a fortnight.

"2. An old labourer of 50 complained of excruciating pains in both legs, twinging and tension in back, feeling of tightness under the ribs, weakness and infirmity in the legs and likewise in the arms, and tickling in the feet. Patient could not walk safely in the dark. The diagnosis showed oscillation when the eyes were shut, distinct ataxy, sense of touch partially destroyed, muscular feeling in the toe and ankle considerably reduced. The patient was then put on to one gramme analgen twice daily. In two hours considerable relief was experienced, the headache ceased, and in the evening the sleep was quieter. Fourteen days later the patient was free from all pain, having taken altogether 30 grammes analgen.

"3. An old merchant of 44, free from lues, had complained for some time of severe pains and weakness in both legs, followed later on by want of feeling and coldness in the soles of the feet and the feet generally, creeping sensation and itching in the back and in the leg, and also pain in the waist. Oscillation when eyes shut, unsteady walk, loss of the sense of touch. The reflex action failed in the legs, but was present in the skin. The patient was treated in a clinical hospital with the faradic pencil for several weeks; the result was satisfactory at first, as regular sleep came on, the pains lessened, and the disturbances in the sensibility came back. This condition did not, however, continue. After some weeks the stinging pains and tightening of the waist reappeared. He now took daily morning and evening one gramme analgen, and noticed in four days considerable improvement. No stinging pains, or feeling of tightness in the waist."

In our own case of locomotor ataxy, attended with very severe tightening pains and unilateral headache, the relief was most remarkable after a few doses of analgen of eight grains each, although the pains were hardly affected by phenazonum, the bromides, exalgen, &c.

We confess to some disappointment in the limited experience we have had of this remedy, especially when we find from the very favourable reports collected by the manufacturers from German sources from which we have quoted typical examples. A further and more extended trial may prove that we have been exceptionally unfortunate in our earlier cases, and we should be glad to receive from practitioners clinical notes on cases in which this drug has been used by them.

